# Medication Use and Costs Among Older Adults Aged 90 Years and Older in Italy

**DOI:** 10.3389/fphar.2022.818875

**Published:** 2022-03-18

**Authors:** Maria Beatrice Zazzara, Agnese Cangini, Roberto Da Cas, Ilaria Ippoliti, Alessandra Marengoni, Andrea Pierantozzi, Elisabetta Poluzzi, Simona Zito, Graziano Onder

**Affiliations:** ^1^ Fondazione Policlinico Universitario A. Gemelli IRCCS, Rome, Italy; ^2^ Agenzia Italiana Del Farmaco, Rome, Italy; ^3^ Pharmacoepidemiology Unit, National Centre for Drug Research and Evaluation, Istituto Superiore di Sanità, Rome, Italy; ^4^ Department of Clinical and Experimental Sciences, Università Degli Studi di Brescia, Brescia, Italy; ^5^ Department of Medical and Surgical Sciences, University of Bologna, Bologna, Italy; ^6^ Department of Cardiovascular and Endocrine-Metabolic Diseases and Aging, Istituto Superiore di Sanità, Rome, Italy

**Keywords:** older adults, medication appropriateness, inappropriate prescribing, centenarians, medication use

## Abstract

Older adults are often affected by multiple chronic conditions and experience geriatric syndromes that may affect the risk/benefit profile of medications. Little is known about the use of such medications in the older population. This article describes medication use and costs in Italian adults aged ≥90 years. Data from the 2019 Pharmaceutical Prescriptions database, concerning data on medications reimbursed by the Italian National Health Service, were analyzed in terms of prevalence and amount of use expressed as defined daily dose/1,000 users (DDD/1,000 users/day), accounting for different age-groups and sex. All individuals aged ≥90 years used at least one medication, with a mean number of 3128 DDD/1,000 users/day corresponding to an annual cost of 683 euros per user. Both use and costs linearly decreased with increasing age, with men accounting for a higher amount of DDD/1,000 users and costs than women across all age-groups. Antihypertensives (1330 DDD/1,000 inhabitants), antiplatelet agents (337 DDD/1,000 inhabitants), medications for peptic ulcer and gastroesophageal reflux (328 DDD/1,000 inhabitants), and lipid-lowering agents (166 DDD/1,000 inhabitants) were the most frequently used medications. We observed a progressive decrease in the usage of the majority of medications with increasing age, with the exception of antibiotics and antipsychotics. Individuals aged ≥90 years used a lower DDD/1,000 users, with an associated decrease in annual costs. The persistent use of preventive medications highlights the potential lack of awareness regarding medication rationalization and guidance for optimizing prescriptions. Our findings highlight the need for further initiatives to improve medications’ appropriateness in these older age-groups.

## Introduction

The proportion of older adults has increased worldwide (United Nations, 2019) and so has the number of medications regularly used by older adults (Onder et al., 2014a; Charlesworth et al., 2015; Gao et al., 2018). Older adults are often affected by multiple chronic conditions, often have complex polypharmacy regimens, and experience geriatric conditions such as cognitive impairment, hearing and vision impairment, sarcopenia, and frailty that can limit the effectiveness, safety, and ability to use several medications (Cherubini et al., 2012; Poudel et al., 2013; Onder et al., 2014a; Onder et al., 2018).

The use of medications in the oldest old—older adults aged 80 years and older—adds further complexity, due to changes in the risk/benefit profile of pharmacologic treatments (Onder et al., 2014a; Charlesworth et al., 2015; Gao et al., 2018). Treatment adherence might be limited due to the presence of cognitive and functional deficits. Life expectancy may be shorter than the *time until benefit* of medications frequently used for primary or secondary prevention (Onder et al., 2014a; Onder et al., 2018). In addition, the use of multiple medications may trigger the onset of and worsen the symptoms of geriatric syndromes, such as falls (Dhalwani et al., 2017) and delirium (Aloisi et al., 2019), with a significant impact on the quality of life of the oldest old. Furthermore, medication metabolism is often altered by kidney and liver disorders, leading to an increased risk of adverse drug reactions, hospitalization, medication-related morbidity and mortality (Cherubini et al., 2012; Pérez et al., 2018; Fralick et al., 2020; Zazzara et al., 2021), and healthcare costs (Watanabe et al., 2018). Finally, older multimorbid and frail individuals are frequently excluded from clinical trials (Poudel et al., 2013; Aloisi et al., 2019), with scarce evidence and guidelines relevant for this population (Dhalwani et al., 2017; Zazzara et al., 2021).

In the absence of high-quality evidence and recommendations, physicians often face the onerous task of evaluating risks and benefits of pharmacological treatments (Onder et al., 2016). In this study, we aim to outline national characteristics of medication use and relevant annual costs in Italian older adults aged 90 years and older and highlight possible differences and similarities with adults aged 70–79 and 80–89 years. These findings may be relevant for identifying ways to improve the prescribing process and deprescribing and to formulate guidance on pharmacological treatments for older adults.

## Methods

### Italian Pharmaceutical Reimbursement System

In Italy, the costs of care for older people are largely covered by the National Health Service [Servizio Sanitario Nazionale (SSN)], based on universal entitlement. The SSN covers costs of pharmacologic treatment for most diseases, providing universal pharmaceutical coverage to the whole population. The conditions of the reimbursement system are established at a national level. Costs of medications are the same all year long and across Italian regions. Reimbursed drugs include essential medications that are proven to be effective for the treatment of acute or chronic diseases (i.e., antihypertensive drugs, antibiotics, hypoglycemic agents, antibiotics, antidepressants, antiplatelet agents, and anticoagulants.). Non-reimbursed drugs include non-essential medications that can be dispensed to citizens with or without a medical prescription and over-the-counter medications (Onder et al., 2014b; The Medicines Utilisation Monitoring Centre, 2021).

### Data Analysis

Data were extrapolated from the Pharmaceutical Prescriptions database (also known as the Italian Health Insurance Card database) that includes anonymized patient-level data on medications prescribed and dispensed by community pharmacies and reimbursed by Italian SSN in the Italian population (The Medicines Utilisation Monitoring Centre, 2021). Information on each drug package, identified via package unique identifier codes and the fifth level Anatomical Therapeutic Chemical (ATC) classification (World Health Organization, 2021), was tracked at individual level in an encrypted format. Based on these data from the Pharmaceutical Prescriptions database, the Osservatorio Nazionale sull'Impiego dei Medicinali “OsMed” (Medicines Utilization Monitoring Centre), an organ of the Italian Medicines Agency (AIFA), publishes an annual report on consumption and expenditure of medications supplied by the SSN and changes over time and across different Italian regions (Italian Medicines Agency, 2021; The Medicines Utilisation Monitoring Centre, 2021). This report aims to facilitate the circulation and dissemination of healthcare-related public information. Article number 50 of Italian Law 24 November 2003, n. 326, with regard to the monitoring of healthcare expenditure and appropriateness of medical prescription, ensure the publication of these data (Gazzetta Ufficiale della Repubblica Italiana, 2003).

We conducted a descriptive analysis of data on patients aged 90 years and older, with an overview of differences and similarities with patients aged 70–79 and 80–89 years. Data were analyzed in terms of prevalence and amount of use expressed as defined daily dose per 1,000 users (DDD/1,000 users per day) and reported accounting for different age-groups, sex, and Italian regions, and in terms of both DDD per 1,000 inhabitants per day and prevalence of use for different pharmaceutical classes of medications. The defined daily dose (DDD) is a technical unit of measurement created to address drug consumption with the aim of reducing intraregional and international variabilities and represents the average maintenance dose per day of a certain medication in adult subjects, in relation to the main therapeutic indication of the drug (therefore, it is a standard unit and not the recommended dose for the single patient) (World Health Organization, 2021).

We initially considered the prevalence of medication use across three macro age-groups: 70–79, 80–89, and 90 years and older. Prevalence was calculated by dividing the number of individuals receiving at least one medication in 2019 by the total number of Italian individuals in that age-group according to the Italian National Institute of Statistics in January 2019 [70–79 years, n = 5,928,218 (9.9% of the whole population); 80–89 years, n = 3,530,515 (5.9%); 90 years or older, n = 765,773 (1.3%)] (Istituto Nazionale di Statistica, 2021a). We then focused our analysis on characteristics of use per pharmaceutical class of medications within the age-group of oldest individuals aged 90 years or older, which was further subcategorized into three age-groups: 90–94, 95–99, and ≥100 years. The number of medications was determined by the number of medications prescribed and dispensed in 2019 for each user using fifth level ATC codes, with each individual receiving at least one prescription in 2019. The mean number of DDD per 1,000 users (inhabitants) per day was calculated by dividing the total number of DDDs prescribed and dispensed during 2019 for individuals in each age-group by the total number of adults in the Italian population in that age-group. Results were then divided by 365 days and reported per 1,000/users (inhabitants)/day. The prevalence of use for each pharmaceutical class of medications was calculated by dividing the number of individuals in 2019 receiving at least one medication within a specific pharmaceutical class of medications by the total number of Italian individuals in that age-group (90–94; 95–99, and ≥100 years).

As additional analysis, we estimated variation in medication use as measured by the mean number of DDDs/1,000 users per day, across the 21 Italian regions and autonomous provinces. The costs were calculated based on gross expenditure on the medication on the Italian market (Italian Medicines Agency, 2021). Annual costs per user were calculated by dividing the overall costs of medications prescribed and dispensed during 2019 in individuals in each age-group by the total number of individuals receiving at least one medication during 2019 in the same age-group.

## Results

Data on the trend of medication use in individuals aged 70–79 years, 80–89 years, and 90 years or older are shown in Table 1. Individuals aged 90 years or older used at least one medication and received a mean number of 3128 DDD/1,000 users/day corresponding to an annual cost of 683 euros per user. Individuals aged 80–89 years had a similar prevalence of use but used a substantially higher amount of medications (DDD/1,000 users per day = 3,677, +18%) and were responsible for higher annual costs per user, while individuals aged 70–79 years had a slightly lower prevalence of use (97%), used 3189 DDD/1,000 users per day similarly to those aged 90 years and older, and had intermediate annual costs per user. Men had higher usage and were responsible for higher costs than women, across all age-groups. The median number of medications per day was seven in both men and women aged 70–79 years and eight in both men and women aged 80–89 and 90 years and older. The proportion of women use ≥5 or ≥10 medications was higher than that of men in the age-group 70–79 years (70 vs. 68% and 30 vs. 28%, respectively) but lower in those aged 90 years or older (76 vs. 79% and 35 vs. 39%).

**TABLE 1:** Medication use by sex and age-group (2019).

	Total	Men	Women
70–79 years			
DDD/1,000 users per day	3,189	3,494	2,932
Prevalence of use (%)	97	97	97
Annual cost per user (Euros)	670	733	617
Number of medications (median—25^th^–75^th^ percentile)	7 (4–10)	7 (4–10)	7 (4–10)
Users with 5+ medications (%)	69	68	70
Users with 10+ medications (%)	29	28	30
80–89 years			
DDD/1,000 users per day	3,677	3,981	3,472
Prevalence of use (%)	100	100	100
Cost per user (Euros)	805	890	748
Number of medications (median—25^th^–75^th^ percentile)	8 (5–12)	8 (5–12)	8 (5–12)
Users with 5+ medications (%)	79	79	79
Users with 10+ medications (%)	38	38	38
≥90 years			
DDD/1,000 users per day	3,128	3,483	2,984
Prevalence of use (%)	100	100	100
Cost per user (Euros)	683	802	634
Number of medications (median—25^th^–75^th^ percentile)	8 (5–11)	8 (5–12)	8 (5–11)
Users with 5+ medications (%)	76	79	76
Users with 10+ medications (%)	36	39	35

**FIGURE 1:**
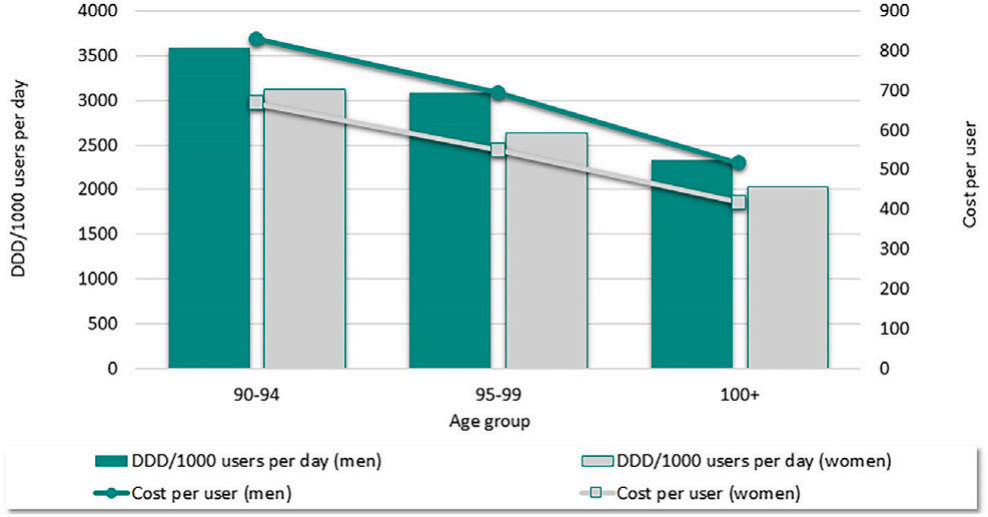
Mean number of DDD/1,000 users per day and cost per user by sex and age-group (2019).

Figure 1 shows that among individuals aged ≥90 years, the number of DDD/1,000 users and annual costs linearly decreased with increasing age, with a substantial reduction in medication use and annual costs among centenarians. Differences between men and women were consistent across age-groups, with men taking a higher number of medications per day (3587 DDD/1,000 users in men aged 90–94; 3122 DDD/1,000 in women aged 90–94) and having a higher overall expenditure (829 euro annual cost per male user aged 90–94 versus 667 euro annual cost per female user aged 90–94). In addition, we observed variability across the 21 Italian regions and autonomous provinces in terms of DDD/1,000 users per day, with values ranging from 2849 DDD/1,000 users to 3467 DDD/1,000 users per day with a difference of 32% (Supplementary Figure S1).

Consumption of DDD/1,000 inhabitants/day per pharmaceutical class of medications according to the three subcategories of oldest adults aged 90–94, 95–99, and≥100 years and sex is presented in Table 2. Antihypertensives were the most frequently used medications (1330 DDD/1,000 inhabitants per day) among Italian older adults aged 90 years and older, followed by antiplatelet agents, medications for peptic ulcer and gastroesophageal reflux disease (GERD), and lipid-lowering agents (ranged between 337 and 166 DDD/1,000 inhabitants per day). With increasing age, we observed a significant reduction in the use of most classes of medications analyzed, with the exception of antibiotics and antipsychotics that showed an increase in DDD/1,000 inhabitants per day, especially in the ≥100 years of age-group. We observed a substantial reduction in the use of antihypertensive agents with increasing age (1361 DDD/1,000 inhabitants per day for the 90–94 age-group, 1241 DDD/1,000 inhabitants per day for the 95–99 age-group, and 985 DDD/1,000 users per day for the group aged ≥100). The use of antiplatelet agents was consistent across all age-groups of adults aged ≥90 years with only small differences between men (382 DDD/1,000 inhabitants per day) and women (321 DDD/1,000 inhabitants per day). The use of medications for peptic ulcer and GERD and lipid-lowering agents demonstrated a progressive reduction of usage across the three different age-groups with a higher consumption level in men than in women for both classes of medications. Antiparkinsonian and antidementia medications showed a significant reduction between the three subcategories of older individuals, especially among centenarians. Table 2 also shows that medications for genito-urinary disorder were used mostly by men (493 DDD/1,000 inhabitants per day in men compared to one DDD/1,000 inhabitants per day in women). Conversely, medications for osteoporosis treatment were more frequently used by women (108 DDD/1,000 inhabitants per day) than men (44 DDD/1,000 inhabitants per day).

**TABLE 2:** Consumption (DDD/1,000 inhabitants per day) by sex and age-group (2019) for pharmaceutical classes.

	Men				Women				Total			
	90-94	95-99	100+	Total	90-94	95-99	100+	Total	90-94	95-99	100+	Total
Antihypertensives	1,416	1,293	950	1,391	1,339	1,226	992	1,308	1,361	1,241	985	1,330
Antiplatelet agents	379	398	377	382	319	331	310	321	336	345	320	337
Medications for peptic ulcer and GERD	350	360	329	351	323	313	273	320	331	323	281	328
Lipid-lowering agents	239	148	63	223	162	93	44	145	184	105	47	166
Medications for genito-urinary disorders	496	486	404	493	1	1	1	1	142	106	63	133
Antidepressants	93	87	72	92	139	115	80	133	126	109	79	122
Antidiabetics	146	104	64	138	118	87	61	111	126	91	61	118
Anticoagulants	139	115	84	135	108	95	71	104	117	100	73	112
Medications for per asthma and COPD	162	155	127	160	81	78	67	80	104	95	76	102
Medications for eye disorders	121	116	110	120	85	78	56	83	95	86	64	93
Medications for osteoporosis	44	44	42	44	116	85	55	108	95	76	53	91
Thyroid medications	21	18	13	21	47	37	24	44	39	33	22	38
NSAIDs	33	32	32	33	38	32	25	36	37	32	26	35
Antibiotics	36	44	54	38	28	33	36	29	30	35	39	31
Pain medications	23	21	17	22	34	30	21	33	31	28	20	30
Antiparkinsonian medications	27	17	11	25	17	11	7	16	20	13	7	18
Antiepileptics	16	14	12	16	15	13	9	14	15	13	10	15
Antipsychotics	10	13	13	11	14	17	15	15	13	16	15	14
Antidementia medications	12	5	2	11	14	6	2	12	14	6	2	12

GERD = gastroesophageal reflux disease.

COPD = chronic obstructive pulmonary disease.

NSAIDS = non-steroidal anti-inflammatory drug.

The prevalence of use per pharmaceutical class of medications according to subcategories of oldest adults aged (90–94, 95–99, and ≥100 years) and sex is shown in Supplementary Table S1.

## Discussion

In this study, we showed that among older adults aged 90 years and over, consumption of medications progressively decreases with increasing age, with an associated decrease in overall annual costs. Although the prevalence and median number of medications per day appear comparable to adults aged 80–89 years, the reduction in the amount of medications used (as measured by mean number of DDD/1,000 users per day) was particularly significant, especially among centenarians.

The hypothesis that our results could only be related to a change in the number of users per day, in a country with such high proportions of oldest old as Italy (Istituto Nazionale di Statistica, 2021), seems a bit simplistic and might undermine important nuances of this complex population.

The gradual reduction of medication burden after 90 years of age might be due to physiological changes in pharmacodynamics and pharmacokinetics that occur with aging that require a mandatory adjustment of the dosage, determining a decrease in DDD/1,000 users per day (Zazzara et al., 2021). Our observation could relate to a more sophisticated phenomenon referred to as the “*healthier survivor effect*”—the possibility of a natural selection of healthier subjects more resistant to traditional risk factors (Robins, 1986; Evert et al., 2003; Hadley and Rossi, 2005; Hagberg and Samuelsson, 2008; Onder et al., 2016) that might have delayed or eluded the onset of common diseases and fatal illness (Evert et al., 2003).

Furthermore, the reduction in the amount of medications could be explained by a different attitude of physicians toward the prescribing process, with increased attention to avoiding inappropriate prescriptions (Cherubini et al., 2012; Onder et al., 2014b). For example, we have outlined a significant decrease in the use of lipid-lowering medications, probably related to an increased awareness among clinicians of their relatively reduced beneficial effect in primary prevention (Armitage et al., 2019).

However, we observed a frequent use of medicines prescribed for the treatment and primary and secondary prevention of cardiovascular events, such as antihypertensives and antiplatelet agents, whereas the use of preventive treatment in the older population may not always seem appropriate when life expectancy versus time to benefit of the medications is taken into consideration.

If true that inappropriate prescriptions involve both unnecessary or omitted treatments and age alone cannot be a detriment to a new prescription when needed, especially if in the absence of any contraindications (Cherubini et al., 2012), at the same time the use of some specific classes of medications in the very old population raises some concerns. Despite extensive literature addressing the role of acetylsalicylic acid in older adults and suggesting an unbeneficial role in several cases (O’Sullivan, 2019), we still found a high prevalence of antiplatelet agent use, likely prescribed for secondary prevention in the examined population. Particular concern also emerges from the data on GERD medication, the prolonged use of which is associated with important adverse events in older adults (Maes et al., 2017). Concern also derives from the data on the use of medications for osteoporosis treatment. The evidence of benefit versus harm over longer periods of treatment in frail multimorbid individuals is scarce, and therefore, a continuous utilization among centenarians appears inappropriate (National Guideline Centre, 2016; Onder et al., 2018). The persistent use of preventive medications might reflect a lack of awareness of physicians toward medication reconciliation, review, and deprescribing (Crisafulli et al., 2021) in the oldest old. This highlights the need for further guidance to improve appropriate prescribing and identify potentially inappropriate medications and potential omissions, thus avoiding any possible age-related bias (Onder et al., 2018; Zazzara et al., 2021). Furthermore, we highlighted a significant difference in medication use between men and women that likely indicates a higher burden of chronic disease in men at older age (Prince et al., 2015).

Finally, we outlined a regional variability of drug utilization across Italy that could reflect different regional demographics, regional regulations, or different distribution of chronic diseases (Onder et al., 2018; Zazzara et al., 2021). While evaluating reasons for this national variability is behind the scope of this work, it could reflect the lack of precise clinical guidelines on prescribing in this population.

### Strengths and Limitations

In this study, we have analyzed data of the entire Italian population aged 90 years and older and provided important insights into medication utilization. Italy is one of the countries with the oldest population worldwide, with more than seven million people aged 75 years and older and 765 thousand aged over 90 in 2019 (Istituto Nazionale di Statistica, 2021b). These data, though not generalizable to populations from other countries, frame a picture of a country with a high proportion of older individuals and reflect the difficulties and issues of constructing precise prescription guidelines for the oldest adults.

Nonetheless, this study has several limitations. We conducted a descriptive analysis on a national database that collects prespecified information, and possible confounders might have been undermined. Due to the nature of the data, we were unable to address differences in medication use according to important geriatric syndromes, such as frailty and cognitive and physical impairment. The analysis relied on data from an administrative database and information on the diagnosis for which medications were dispensed was not available. Eliciting whether the indication for a medication was appropriate is thus not possible. Also, the analysis did not assess data on compliance and actual intake of the medications, particularly important in case of individuals with cognitive impairment or neglected care.

Furthermore, data on reimbursed medications exclude unfilled prescriptions and non-reimbursed medications such as benzodiazepines or phytotherapics or over-the-counter medications. This might have led to an underestimation of the mean number of medications, consumption, and expenditure. Finally, the annual analysis may have included individuals who died within the year and the difference in medication use across the three different macro age-groups (70–79; 80–89; ≥100) might have been influenced by a different mortality rate. Therefore, the reduction of medication utilization in the older decades might be a partial reflection of an increased mortality or a shorter period of observation.

## Conclusion

In our study, we described characteristics of medication use and related costs in the oldest individuals aged ≥90 years, and with regard to those aged 70–79 and 80–89 years. There are limited data from clinical trials and guidance relevant for these older age-groups. Our study highlights the need for evidence to improve medication use in the oldest old and allow physicians to feel more confident when prescribing for older adults.

A targeted—yearly—review of medication regimens is strongly advised to avoid utilization of medications that can become redundant or even dangerous for the vulnerable older population. The inclusion of older adults in clinical trials will help generate an evidence base for the use of medications in older adults. Our findings are helpful to plan and implement interventions aimed at improving the appropriateness of medication use, influencing policy makers, and reducing national variability.

## Data Availability

The datasets presented in this study can be found in online repositories. The names of the repository/repositories and accession number(s) can be found below: Raw data on consumption and expenditure of medications at a national and regional level are published every year in the Open data by the AIFA’s Medicines Utilisation Monitoring Centre (OsMed). https://www.aifa.gov.it/en/dati-aifa.
