# Supplementary material for: Medication Use and Costs Among Older Adults Aged 90 Years and Older in Italy
Source: Front Pharmacol. 2022 Mar 18;13:818875. doi: 10.3389/fphar.2022.818875 (PMC8971522; doi:10.3389/fphar.2022.818875)
Supplement: Supplementary file 1 [file DataSheet1.PDF]

Supplementary Figure 1. Consumption (DDD/1000 users per day) by region (2019)

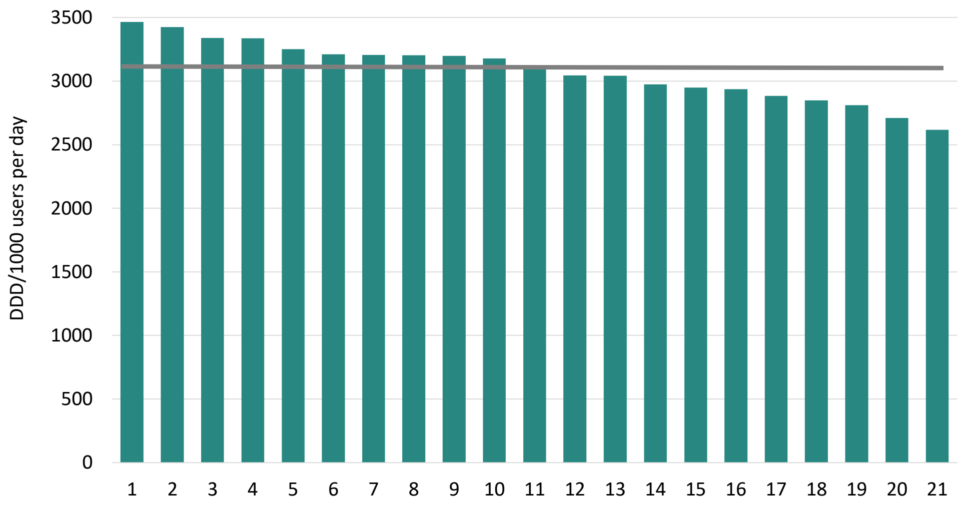

Supplementary Table 1. Prevalence of use (%) by sex and age group (2019) for pharmaceutical groups.

	Men				Women				Total			
	**90-94**	**95-99**	**100+**	**Total**	**90-94**	**95-99**	**100+**	**Total**	**90-94**	**95-99**	**100+**	**Total**
Antihypertensives	100	100	100	100	93	98	95	94	95	99	96	96
Antiplatelet agents	54	59	60	55	46	50	49	47	48	52	51	49
Medications for peptic ulcer and GERD	70	75	74	71	63	65	62	64	65	67	64	66
Lipid-lowering agents	33	22	13	31	25	16	9	23	28	18	10	25
Medications for genito-urinary disorders	50	52	46	50	<0.5	<0.5	<0.5	<0.5	14	12	7	14
Antidepressants	22	25	24	22	27	28	24	27	26	27	24	26
Antidiabetics	20	17	13	19	17	14	11	16	18	15	12	17
Anticoagulants	35	33	28	34	29	29	24	29	31	30	25	31
Medications for per asthma and COPD	30	34	37	31	21	23	25	21	23	25	27	24
Medicines for eye disorders	13	13	12	13	9	9	7	9	10	10	8	10
Medications for osteoporosis	23	24	23	23	41	35	28	40	36	33	27	35
Thyroid medications	6	6	5	6	12	11	8	12	10	10	7	10
NSAIDs	27	27	28	27	28	25	22	27	28	26	23	27
Antibiotics	69	81	96	71	60	69	77	62	62	71	80	64
Pain medications	19	19	18	19	23	22	18	22	22	21	18	21
Antiparkinsonian medications	5	5	3	5	4	3	2	4	4	4	3	4
Antiepileptics	5	5	6	5	5	5	5	5	5	5	5	5
Antipsychotics	11	15	16	11	12	16	16	13	12	15	16	12
Antidementia medications	2	1	1	2	2	1	0	2	2	1	1	2
